# 

**Published:** 1887-11-26

**Authors:** 


PRICE ONE PENNY.

No. 61, Vol. III—November 26th, 1887.

**Registered at the General Post Office as a Newspaper.**

Per Annum, 6s. 6d., post free.

Monthly Parts, 6d.; post free, 7½d.

Ditto per Ann., 7s. 6d. post free.

